# Supplementary material for: Probing the temperature of supported platinum nanoparticles under microwave irradiation by in situ and operando XAFS
Source: Commun Chem. 2020 Jul 3;3:86. doi: 10.1038/s42004-020-0333-y (PMC9814256; doi:10.1038/s42004-020-0333-y)
Supplement: Supplementary file 1 — Supplementary Information [file 42004_2020_333_MOESM1_ESM.docx]

**Supplementary Information: Probing the temperature of supported platinum nanoparticles under microwave irradiation by *in situ* and *operando* XAFS**

Taishi Ano^1^, Shuntaro Tsubaki^1,2^*, Anyue Liu^1^, Masayuki Matsuhisa^1^, Satoshi Fujii^1,3^, Ken Motokura^1^, Wang-Jae Chun^4^, Yuji Wada^1^*

^1^ School of Materials and Chemical Technology, Tokyo Institute of Technology, E4-3, 2-12-1, Ookayama, Meguro-ku, Tokyo 152-8552, Japan
^2^ PRESTO, Japan Science and Technology Agency (JST), 4-1-8 Honcho, Kawaguchi, Saitama 332-0012, Japan

^3^ Department of Information and Communication Systems Engineering, Okinawa National College of Technology, 905 Henoko, Nago-shi, Okinawa, 905-2192, Japan.

^4^ Graduate School of Arts and Sciences, International Christian University, 3-10-2 Osawa, Mitaka-shi, Tokyo, 181-8585, Japan

**Supplementary Note 1: Characterization of Supported Pt Nanoparticles.**

The dielectric properties of the samples before and after drying were summarized in Supplementary Table 1. The real part of the dielectric constant (relative dielectric permittivity; *ε*’) indicates the ability to store MW energy while the imaginary part (dielectric loss; *ε*’’) indicates the MW energy loss. The ratio of *ε*’’/*ε*’ is dielectric loss tangent (tan *δ*) that describes the extent of the microwave (MW) absorption by the materials. The dielectric constants were obtained before and after drying to consider the contribution of water adsorption. Al_2_O_3_ support exhibited higher MW absorption ability than SiO_2_ support owing to the defect vacancy of γ-Al_2_O_3_ structure. The tan *δ* values of all samples were decreased by drying.

**Supplementary Table 1.** Dielectric properties of catalysts measured by the perturbation method at 2.45 GHz.

| Sample | Before drying | | | After drying | | | Weight loss by  TG (%) |
| --- | --- | --- | --- | --- | --- | --- | --- |
|  | *ε*’ | *ε*’’ | tan *δ* | *ε*’ | *ε*’’ | tan *δ* |  |
| Al_2_O_3_ | 2.25 | 0.180 | 0.080 | 1.51 | 0.029 | 0.019 | - |
| Pt/Al_2_O_3_ | 2.34 | 0.301 | 0.128 | 1.50 | 0.026 | 0.017 | 10.2 |
| SiO_2_ | 1.22 | 0.022 | 0.018 | 1.11 | 0.004 | 0.003 | - |
| Pt/SiO_2_ | 1.74 | 0.043 | 0.025 | 1.28 | 0.002 | 0.002 | 4.4 |

Moreover, the extent of decrease was dependent on the amount of absorbed water measured by TG (Supplementary Fig. 1). γ-Al_2_O_3_ contains a lot of adsorbed water, which bring high *ε*’ and *ε*’’. Moreover, in dry conditions, the defects form dipoles and also bring slightly higher *ε*’’ in the γ-Al_2_O_3_ support compared to that in the SiO_2_ support. In the dry conditions, Pt deposition did not significantly change dielectric property, however, the present complex dielectric constant only indicates the bulk property. The local dielectric property of Pt nanoparticles cannot be evaluated by this method. Then, it is indispensable to develop a new method to evaluate the nano-sized local heating under MW irradiation.

MW heating profiles were indicated in Supplementary Figs. 2-3. Larger overheating was observed for Pt/Al_2_O_3_ system as compared to the Pt/SiO_2_ system (Supplementary Fig. 2). The extent of overheating of Pt/Al_2_O_3_ decreased at the elevated temperature (Supplementary Fig. 3). The adsorbed water was gradually released, and the overheating became smaller by repeating MW heating, which indicated the adsorbed water was crucial for the large MW heating. In Supplementary Fig. 3c, the overheating was observed again 1 hour after the 1st MW heating due to the fast adsorption of the water on the Pt/Al_2_O_3_ surface.


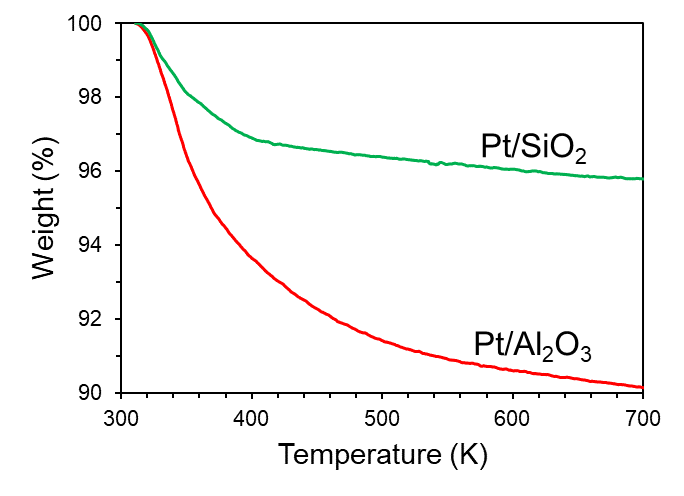


**Supplementary Fig. 1** **TG profiles of Pt/Al_2_O_3_ and Pt/SiO_2_.**


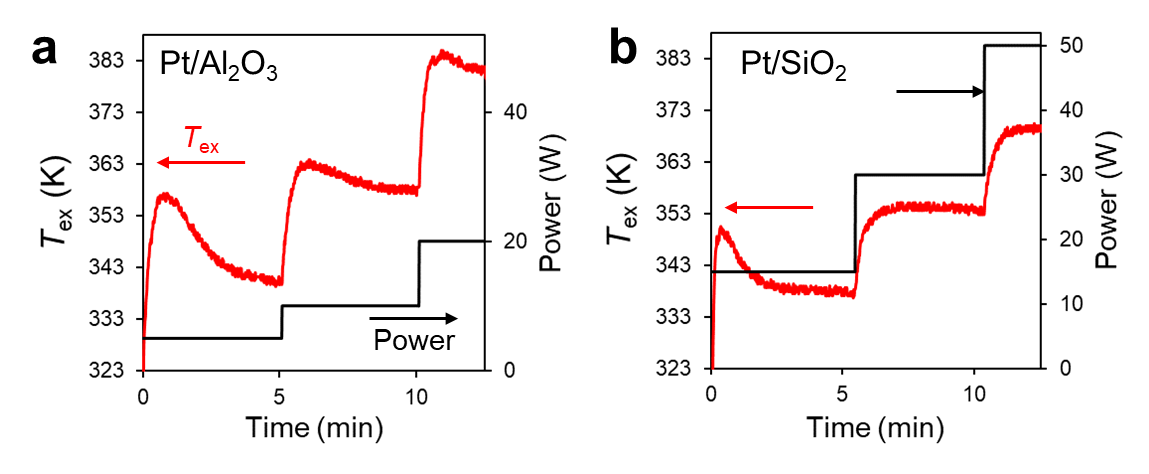


**Supplementary Fig. 2 MW heating profiles of Pt NPs supported catalysts. a** Pt/Al_2_O_3_, **b** Pt/SiO_2_**.**


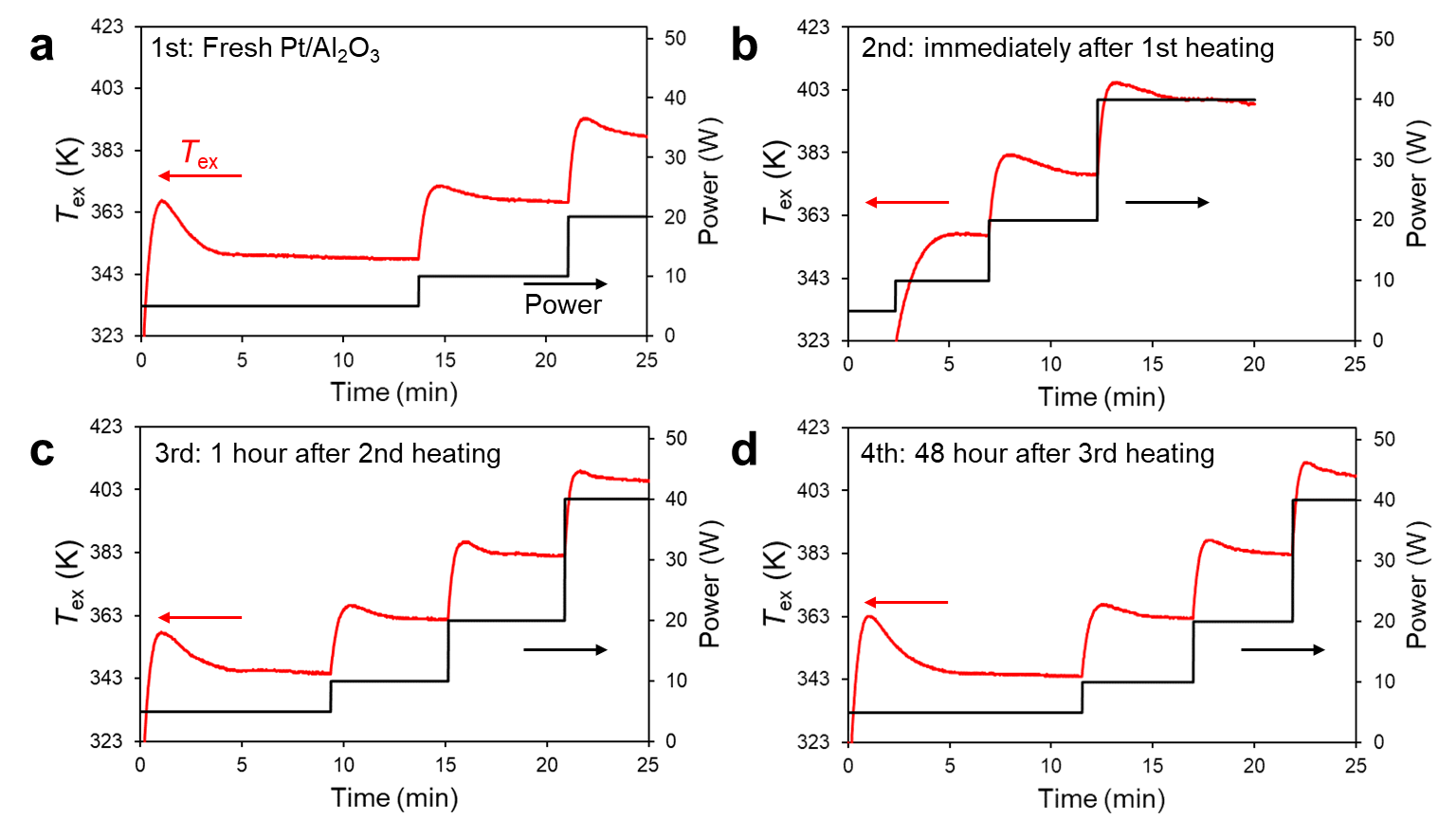


**Supplementary Fig. 3** **Temperature profiles during repeated MW heating of Pt/Al_2_O_3_.** **a** 1st; Fresh sample, **b** 2nd; sample immediately after the 1st heating, **c** 3rd; sample 1 hour after the 2nd heating, **d** 4th; sample 48 hours after the 3rd heating.

**Supplementary Note 2: *In-situ* XAFS spectroscopy for Nano-thermometric Analysis of Supported Pt Nanoparticles.**

XAFS spectra of a packed pellet of Pt/Al_2_O_3_ were obtained by using an *in-situ* XAFS cell (Supplementary Fig. 4a) for conventional heating (CH) condition, or a microwave (MW) set-up (Supplementary Fig. 4b), and the XAFS spectra are shown in Supplementary Fig. 5. The XANES spectra at 298–573 K were consistent, while in the 673 K, the white line intensity increased and shifted to the higher photon energy. The increase in the white line peak intensity attributes to the oxidation of Pt nanoparticles (NPs) (Fig. 8f). The amplitude of EXAFS oscillation gradually decreased as the temperature rises under CH, which was theoretically explained by equation 1 in text. In contrast, the intensity of the EXAFS signal at 368, 378, 392 K decreased drastically under MWs without changes in the XANES spectra. Finally, the XAFS spectra before and after MW were identical, showing the Pt NPs structure did not change during MW heating.

**
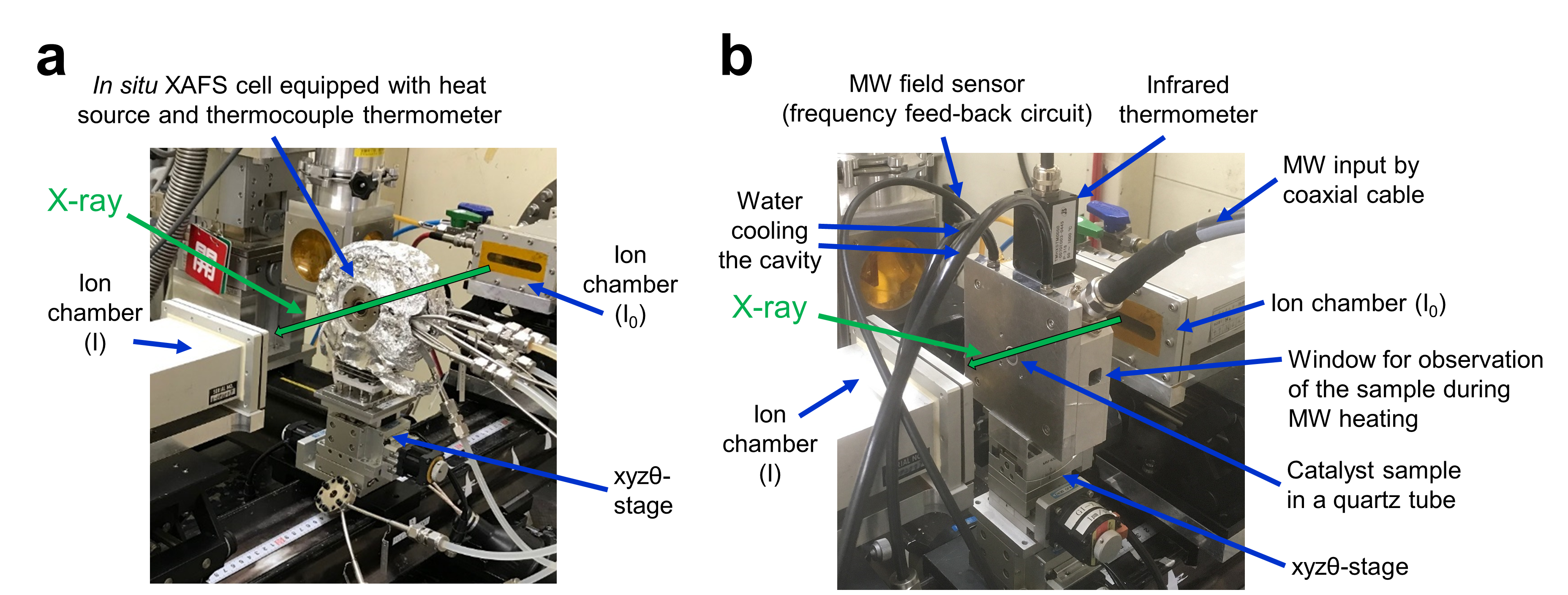
**

**Supplementary Fig. 4 Setup of *in-situ* XAFS for Pt/Al_2_O_3_ catalyst. a** CH and **b** MW heating**.**


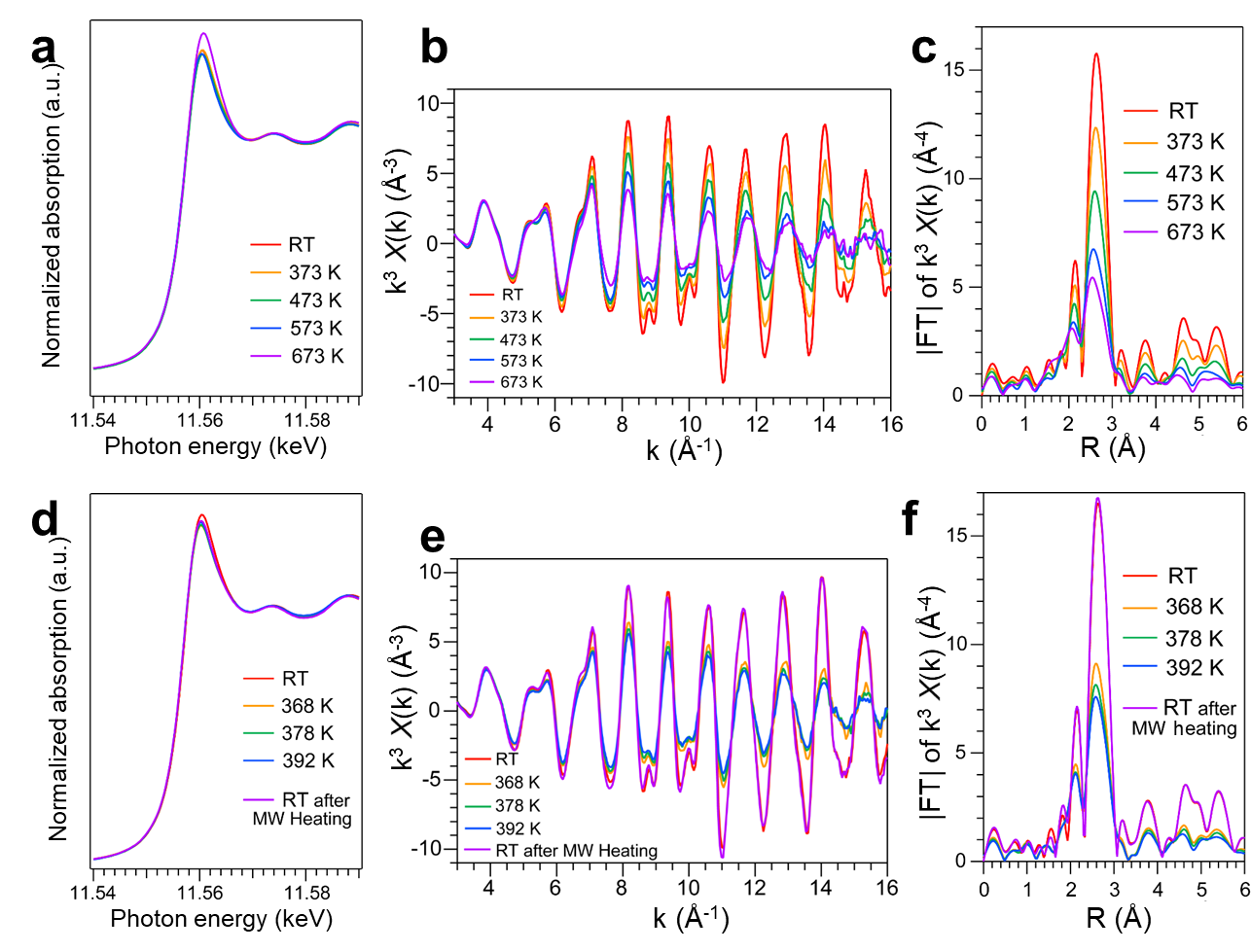


**Supplementary Fig. 5** ***In-situ* XAFS results of Pt/Al_2_O_3_**. **a** XANES, **b** EXAFS, **c** FT-EXAFS spectra under CH, **d** XANES, **e** EXAFS and **f** FT-EXAFS spectra under MW heating.

Supplementary Fig. 6 shows *in-situ* XAFS spectra of Pt foil at 298–673 K. The EXAFS signal and the peak intensity of Pt-Pt bonding at 2.75–2.77 Å in FT-EXAFS spectra decreased at the elevated temperature. Curve fitting analysis is conducted to determine the following parameters of Pt; coordination number (*N*), interatomic distance (*R*), the potential difference of core electrons transition between the object and the reference (Δ*E*_0_) and the Debye-Waller factor (*σ*^2^). The curve-fit values of Pt foil were summarized in Supplementary Table 2 and Supplementary Fig. 7, where the coordination number was fixed to 12 and the *σ*^2^ value at 298 K was set in 0.0036 Å as the reference value, which is used to determine the Δ*σ*^2^ values at 298–673 K under CH. The values of *N*, *R*, and Δ*E*_0_ of structural parameters did not change as the temperature rose, however, the great change was observed in Δ*σ*^2^.


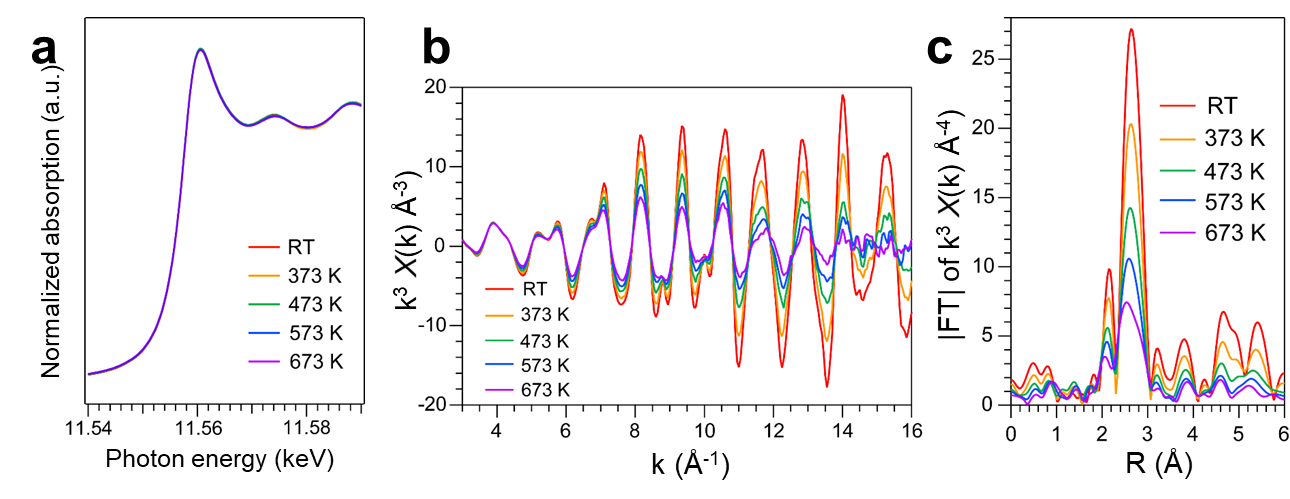


**Supplementary Fig. 6** ***In-situ* XAFS results of Pt foil under CH**. **a** XANES, **b** EXAFS, and **c** FT-EXAFS spectra.

**Supplementary Table 2.** Curve-fit *R*, Δ*E*_0_ and Δ*σ*^2^ values from *in-situ* FT-EXAFS spectra of Pt Foil under CH.*

| *T* (K) | Pt foil (CH) | | | |
| --- | --- | --- | --- | --- |
|  | N | *R* (Å) | Δ*E*_0_ (eV) | Δ*σ*^2^ (Å^2^) |
| 298 | 12 | 2.77 ± 0.01 | 0 ± 2 | 0.00 ± 0.02 |
| 373 | 12 | 2.77 ± 0.01 | -1 ± 2 | 1.16 ± 0.02 |
| 473 | 12 | 2.76 ± 0.01 | -2 ± 2 | 2.64 ± 0.01 |
| 573 | 12 | 2.76 ± 0.01 | -3 ± 2 | 3.80 ± 0.01 |
| 673 | 12 | 2.75 ± 0.01 | -3 ± 2 | 5.43 ± 0.02 |

*N values were fixed to 12. Δ*σ*^2^ values were relative values compared to *σ*^2^ of Pt foil reference. Standard deviations were obtained in curve fitting analysis.


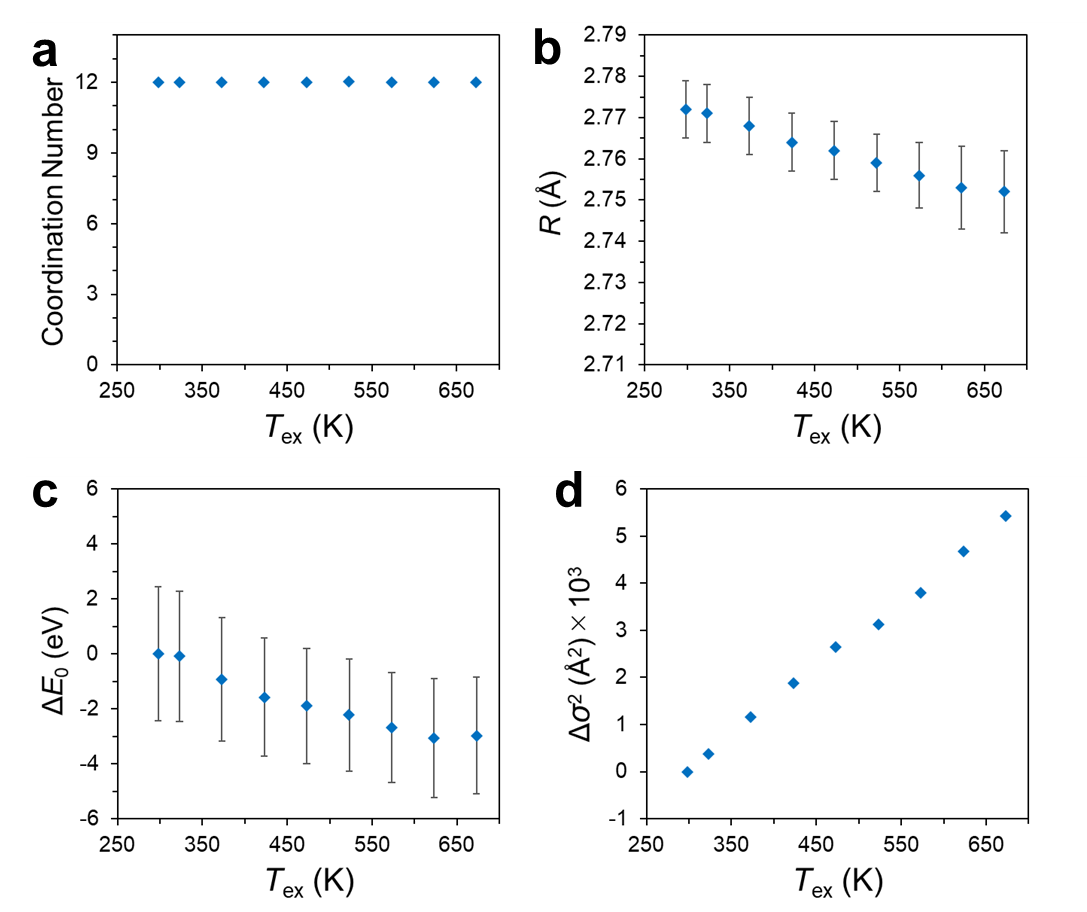


**Supplementary Fig. 7** **Curve-fit parameters from *in-situ* FT-EXAFS spectra of Pt foil under CH.** **a** Fixed Coordination Number: N, **b** *R*, **c** Δ*E*_0_ and **d** Δ*σ*^2^. Δ*σ*^2^ values were relative values compared to *σ*^2^ of Pt foil as reference. Error bars indicate standard deviations obtained in curve fitting analysis.

As for the curve-fit results of Pt/Al_2_O_3_, the values of *N*, *R*, and Δ*E*_0_ were not changed and the great change of Δ*σ*^2^ was observed (Supplementary Table 3 and Supplementary Fig 8). The behavior of Δ*σ*^2^ through the MW heating is shown in Supplementary Fig 9a. The Δ*σ*^2^ values before and after MW were consistent at room temperature. Further, the TEM image of Pt/Al_2_O_3_ after MW (Supplementary Fig 9b) and the size distributions of the Pt NPs before and after MW heating (Supplementary Fig S9c, d) were almost the same. These results also support that there is no structural change in Pt/Al_2_O_3_ by MW heating. In such systems with no structural change, the increase of Δ*σ*^2^ values was attributed to the *σ*_T_ but not to the *σ*_S_, which is explained by equation 2. Therefore, Δ*σ*^2^ values can be used to determine the local temperature of the Pt NPs (*T*_Pt_) in the Pt/Al_2_O_3_ system.

**Supplementary Table 3.** Curve-fit *N*, *R*, Δ*E*_0_ and Δ*σ*^2^ values from *in-situ* FT-EXAFS spectra of Pt/Al_2_O_3_ under CH and MW heating.*

| *T* (K) | Pt/Al_2_O_3_ (CH) | | | | | |  |
| --- | --- | --- | --- | --- | --- | --- | --- |
|  | *N* | | *R* (Å) | Δ*E*_0_ (eV) | Δ*σ*^2^ (Å^2^) |  |  |
| 298 | 9.7 ± 1.7 | | 2.77 ± 0.01 | 0 ± 2 | 1.30 ± 0.10 |  |  |
| 373 | 9.8 ± 1.6 | | 2.76 ± 0.01 | -1 ± 1 | 2.33 ± 0.01 |  |  |
| 473 | 9.7 ± 1.5 | | 2.76 ± 0.01 | -2 ± 1 | 3.46 ± 0.01 |  |  |
| 573 | 10.9 ± 1.7 | | 2.75 ± 0.01 | -2 ± 1 | 5.62 ± 0.01 |  |  |
| 673 | 9.6 ± 1.6 | | 2.75 ± 0.01 | -3 ± 1 | 6.20 ± 0.02 |  |  |
| *T* (K) | Pt/Al_2_O_3_ (MW) | | | | | |  |
|  | *N* | *R* (Å) | | Δ*E*_0_ (eV) | Δ*σ*^2^ (Å^2^) | | |
| 298 | 9.1 ± 1.6 | 2.77 ± 0.01 | | 0 ± 2 | 0.89 ± 0.10 | | |
| 366 | 9.8 ± 1.5 | 2.76 ± 0.01 | | -2 ± 1 | 3.63 ± 0.01 | | |
| 378 | 9.7 ± 1.5 | 2.77 ± 0.01 | | 1 ± 1 | 3.80 ± 0.01 | | |
| 392 | 9.6 ± 1.5 | 2.77 ± 0.01 | | 1 ± 1 | 4.14 ± 0.01 | | |
| 298 | 9.3 ± 1.7 | 2.77 ± 0.01 | | 0 ± 2 | 0.89 ± 0.10 | | |

*Δ*σ*^2^ values were relative ones compared to *σ*^2^ of Pt foil as reference. Standard deviations were obtained in curve fitting analysis.


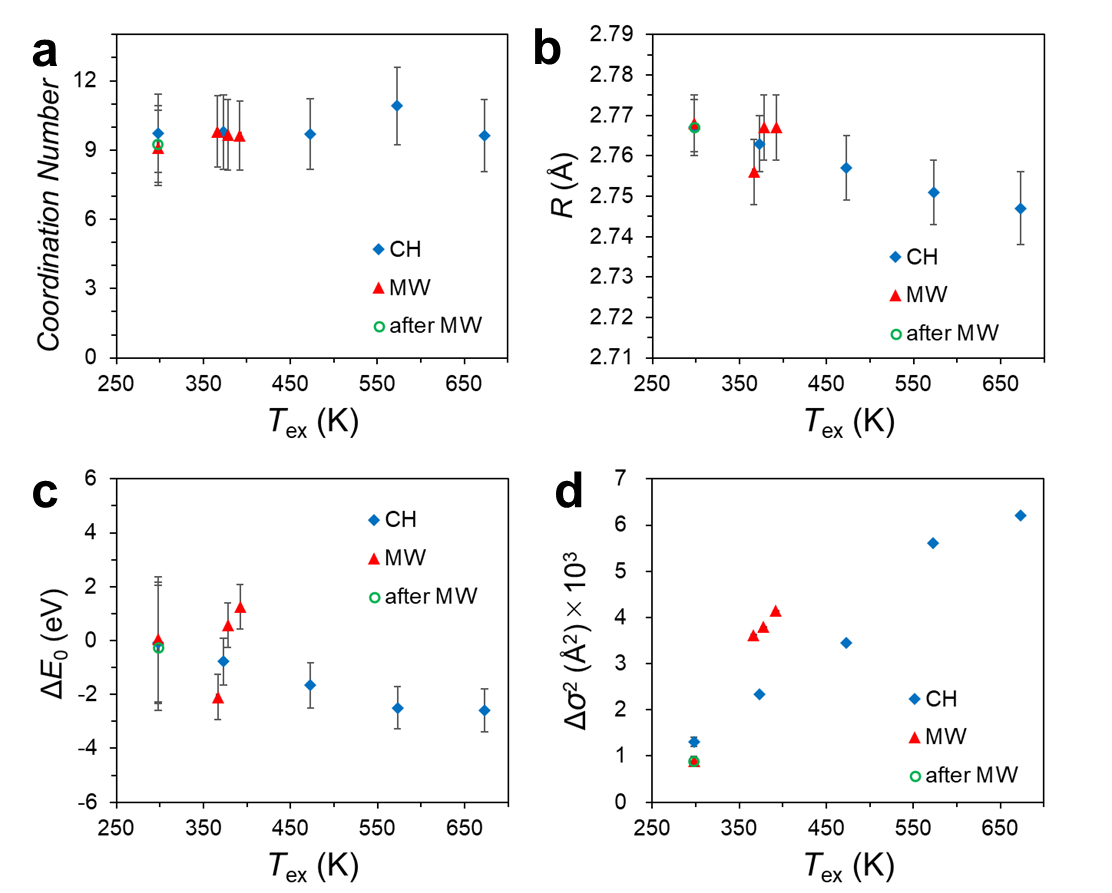


**Supplementary Fig. 8 Curve-fit parameters from *in-situ* FT-EXAFS of Pt/Al_2_O_3_ under CH and MW heating**. **a** Coordination Number: *N*, **b** *R*, **c** Δ*E*_0_ and **d** Δ*σ*^2^. Δ*σ*^2^ values were relative values compared to *σ*^2^ of Pt foil as reference. Error bars indicate standard deviations obtained in curve fitting analysis.


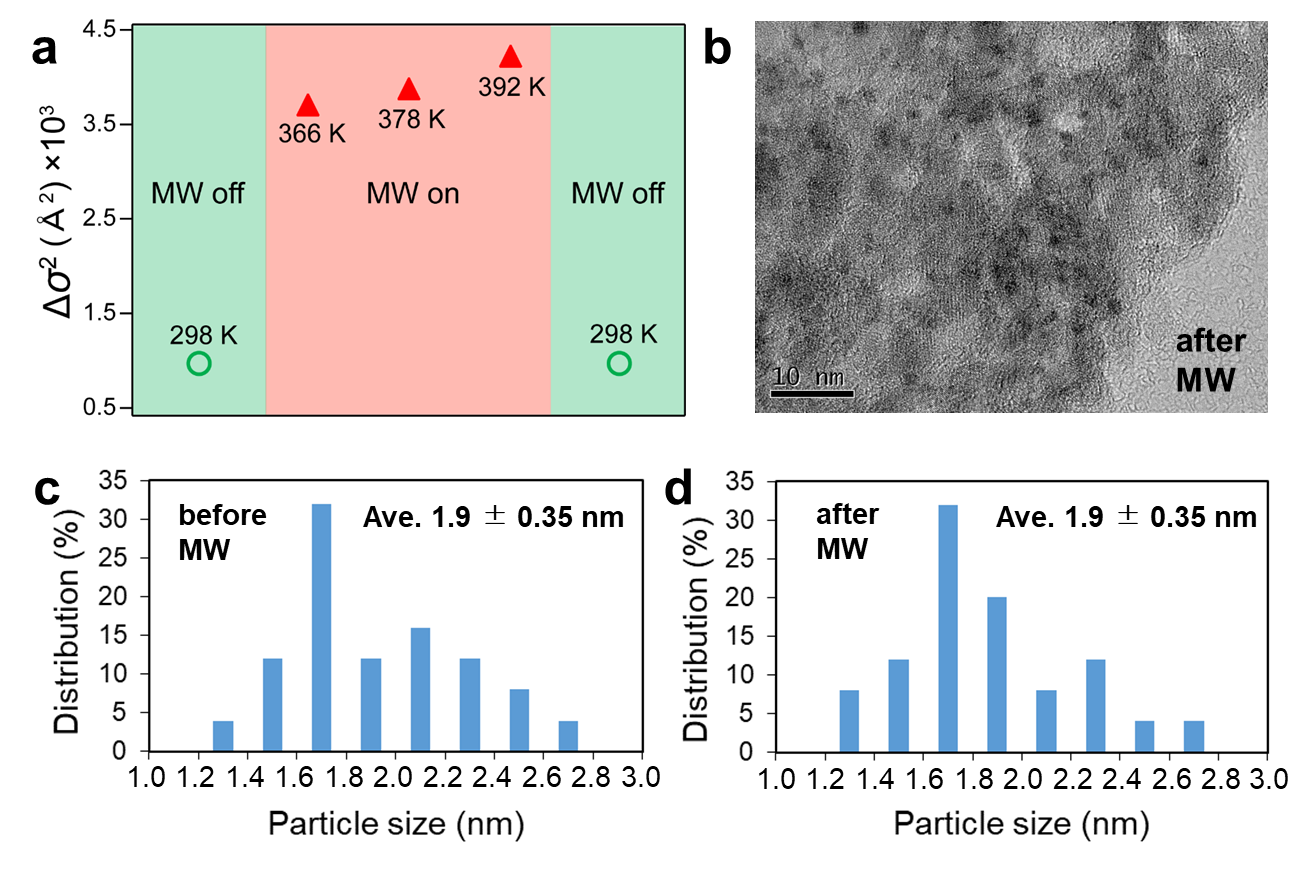


**Supplementary Fig. 9 Comparison of Pt/Al_2_O_3_ structure before and after MW heating. a** Δ*σ*^2^ dynamics over the MW-XAFS experiment. **b** The TEM image of Pt/Al_2_O_3_ after MW heating. **c** The size distribution of the Pt NPs before and **d** after MW heating. Δ*σ*^2^ values were relative values compared to *σ*^2^ of Pt foil as reference.

*In-situ* XAFS spectra of Pt/SiO_2_ and those under 10 mL/min N_2_ or He flows were shown in Supplementary Figs 10,12, respectively. The curve-fit parameters were summarized in Supplementary Fig. 11 and Supplementary Tables 4, 5. As with the Pt/Al_2_O_3_ system, *N*, *R*, and Δ*E*_0_ values did not exhibit a significant change, whereas the Δ*σ*^2^ value was drastically changed. The thermal properties of different gases were summarized in Supplementary Table 6. He gas brought the lower *T*_Pt_ value as shown in Fig. 6 in the text owing to its high thermal conductivity. Moreover, He plasma was observed under high power MW irradiation (Supplementary Fig 13). The generated plasma may consume MW energy and lowered the value of *T*_Pt_ by preventing the MW energy concentration on the Pt NPs.


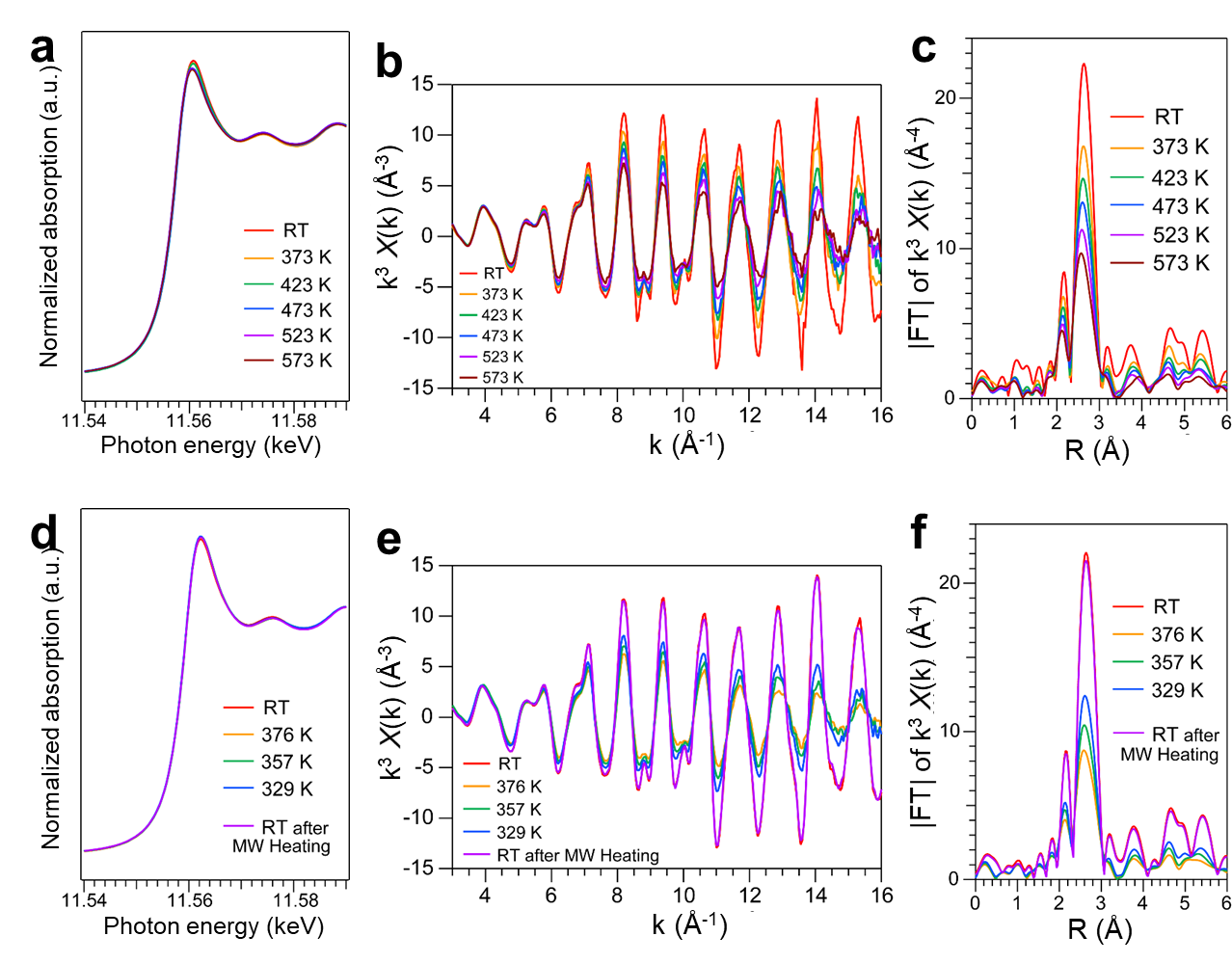


**Supplementary Fig. 10** ***In-situ* XAFS spectra of Pt/SiO_2_.** **a** XANES, **b** EXAFS and **c** FT-EXAFS spectra under CH and **d** XANES, **e** EXAFS, and **f** FT-EXAFS under MW heating.

**Supplementary Table 4.** Curve-fit *N*, *R*, Δ*E*_0_ and Δ*σ*^2^ values from *in-situ* FT-EXAFS spectra of Pt/SiO_2_ under CH and MW heating.***

| *T* (K) | Pt/SiO_2_ (CH) | | | | | |  |
| --- | --- | --- | --- | --- | --- | --- | --- |
|  | *N* | | *R* (Å) | Δ*E*_0_ (eV) | Δ*σ*^2^ (Å^2^) |  |  |
| 298 | 10.5 ± 2.0 | | 2.77 ± 0.01 | 1 ± 2 | 0.37 ± 0.12 |  |  |
| 373 | 10.8 ± 1.9 | | 2.77 ± 0.01 | 0 ± 1 | 1.44 ± 0.01 |  |  |
| 423 | 10.5 ± 1.8 | | 2.76 ± 0.01 | 0 ± 1 | 1.88 ± 0.01 |  |  |
| 473 | 11.0 ± 1.8 | | 2.76 ± 0.01 | -1 ± 1 | 2.64 ± 0.01 |  |  |
| 523 | 11.5 ± 1.8 | | 2.76 ± 0.01 | 2 ± 1 | 3.46 ± 0.02 |  |  |
| 573 | 11.2 ± 1.8 | | 2.76 ± 0.01 | 1 ± 1 | 4.14 ± 0.10 |  |  |
| *T* (K) | Pt/SiO_2_ (MW) | | | | | |  |
|  | *N* | *R* (Å) | | Δ*E*_0_ (eV) | Δ*σ*^2^ (Å^2^) | | |
| 298 | 10.4 ± 2.0 | 2.77 ± 0.01 | | 1 ± 2 | 0.24 ± 0.12 | | |
| 376 | 11.3 ± 1.8 | 2.77 ± 0.01 | | 2 ± 1 | 4.50 ± 0.01 | | |
| 357 | 11.2 ± 1.8 | 2.77 ± 0.01 | | 2 ± 1 | 3.63 ± 0.01 | | |
| 329 | 10.7 ± 1.8 | 2.77 ± 0.01 | | -1 ± 1 | 2.64 ± 0.01 | | |
| 298 | 10.2 ± 1.9 | 2.77 ± 0.01 | | 1 ± 2 | 0.24 ± 0.12 | | |
| - | - | - | | - | - | | |

*Δ*σ*^2^ values were relative ones compared to *σ*^2^ of Pt foil as reference. Standard deviations were obtained in curve fitting analysis.


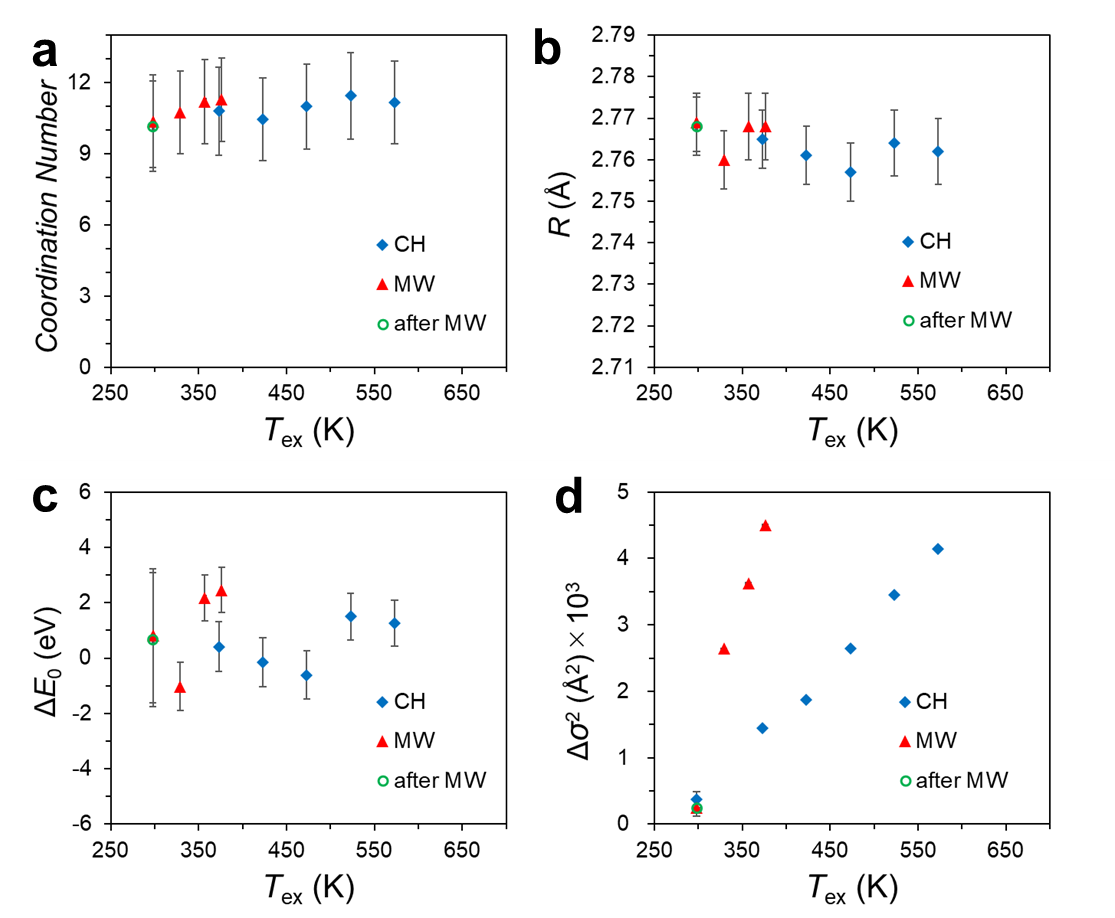


**Supplementary Fig. 11 Curve-fit parameters from *in-situ* FT-EXAFS spectra of Pt/SiO_2_ under CH under MW heating.** **a** Coordination Number: *N*, **b** *R*, **c** Δ*E*_0_ and **d** Δ*σ*^2^. Δ*σ*^2^ values were relative values compared to *σ*^2^ of Pt foil as reference. Error bars indicate standard deviations obtained in by curve fitting analysis.


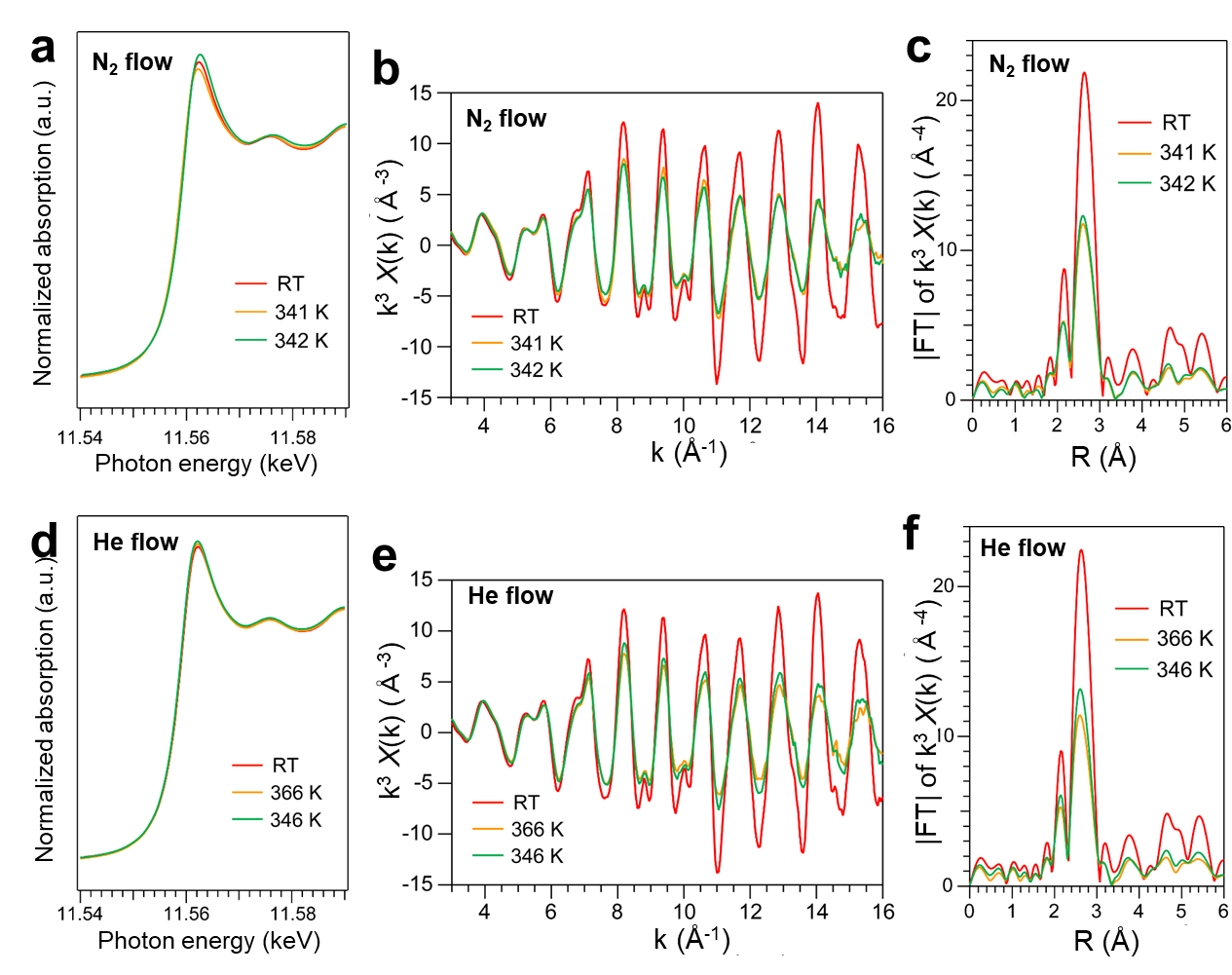


**Supplementary Fig 12 *In-situ* XAFS spectra of Pt/SiO_2_ under MW heating. a** XANES, **b** EXAFS and **c** FT-EXAFS spectra under N_2_ flow, and **d** XANES, **e** EXAFS **f** and FT-EXAFS under He flow.

**Supplementary Table 5.** Curve-fit *N*, *R*, Δ*E*_0_ and Δ*σ*^2^ values from *in-situ* FT-EXAFS spectra of Pt/SiO_2_ under MW heating in N_2_ or He flow conditions.***

| *T* (K) | Pt/SiO_2_ (MW, N_2_ flow) | | | | |  |
| --- | --- | --- | --- | --- | --- | --- |
|  | *N* | | *R* (Å) | Δ*E*_0_ (eV) | Δ*σ*^2^ (Å^2^) |  |
| 298 | 10.4 ± 2.0 | | 2.77 ± 0.01 | 1 ± 2 | 0.37 ±0.12 |  |
| 342 | 11.9 ± 1.9 | | 2.77 ± 0.01 | 3 ± 1 | 3.12 ± 0.01 |  |
| 341 | 11.1 ± 1.8 | | 2.77 ± 0.01 | 3 ± 1 | 3.12 ± 0.01 |  |
| *T* (K) | Pt/SiO_2_ (MW, He flow) | | | | |  |
|  | *N* | *R* (Å) | | Δ*E*_0_ (eV) | Δ*σ*^2^ (Å^2^) | |
| 298 | 10.8 ± 2.0 | 2.77 ± 0.01 | | 0 ± 2 | 0.37 ±0.12 | |
| 366 | 11.2 ± 1.8 | 2.77 ± 0.01 | | 3 ± 1 | 3.29 ± 0.01 | |
| 346 | 10.6 ± 1.8 | 2.76 ± 0.01 | | 0 ± 2 | 2.48 ± 0.01 | |

*Δ*σ*^2^ values were relative ones compared to *σ*^2^ of Pt foil as reference. Standard deviations were obtained in curve fitting analysis.

**Supplementary Table 6.** Thermal properties of air, N_2_, and He gas from the COMSOL Multiphysics database.

| *T* (K) | Thermal conductivity: *κ* [W/(m·K)] | | | Volumetric heat capacity at constant  pressure: *C*_p_ [J/(m^3^·K)] | | |
| --- | --- | --- | --- | --- | --- | --- |
|  | Air | N_2_ | He | Air | N_2_ | He |
| 298 | 0.026 | 0.026 | 0.141 | 0.0118 | 0.0117 | 0.0084 |
| 373 | 0.032 | 0.031 | 0.171 | 0.0094 | 0.0094 | 0.0067 |
| 423 | 0.035 | 0.035 | 0.185 | 0.0084 | 0.0083 | 0.0059 |
| 473 | 0.039 | 0.038 | 0.198 | 0.0076 | 0.0075 | 0.0053 |


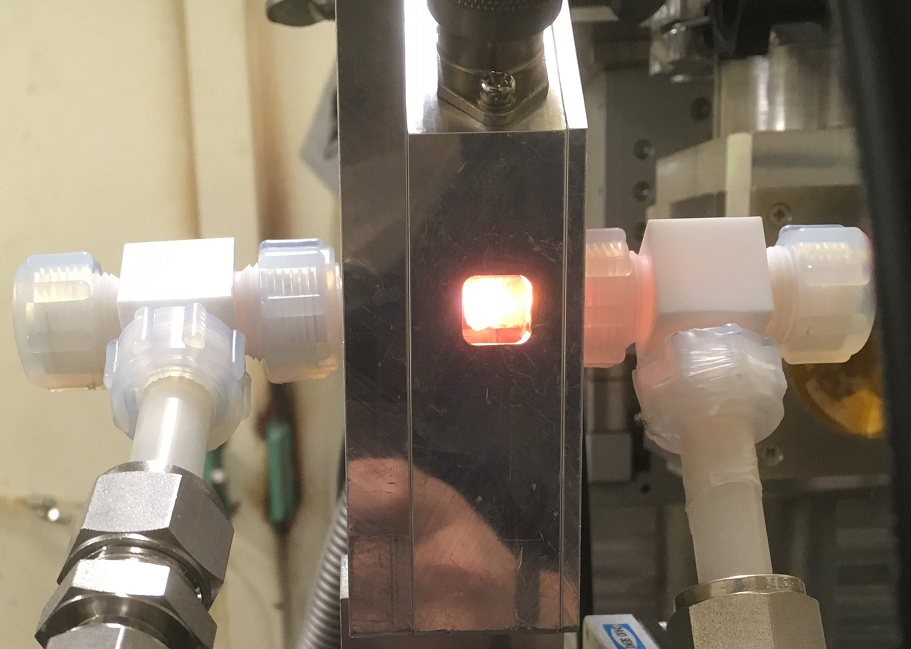


**Supplementary Fig. 13 Plasma formation under 60 W MW heating and He flow in Pt/SiO_2_ system.**

**Supplementary Note 3: Determination of Temperature Distribution in the Catalyst Pellet.**

The temperature distribution in the catalyst pellet by MW heating was determined by a combination of thermography and coupled simulation as indicated in the main text (Supplementary Fig. 14). The temperature difference between catalyst bed side (*T*_ex_) and core temperature (*T*_in_) are indicated in Fig. 3. Supplementary Fig. 15 summarizes the profiles of *T*_ex_ and *T*_in_ values under several MW powers. The simulated distributions of the electric field and the temperature as well as the temperature profile at each point of Pt/Al_2_O_3_ pellet under 18 W MWs were summarized in Supplementary Fig. 16. For instance, after 3 min MW heating, the center of the surface by air was only 8 K lower than the core of the pellet (Supplementary Fig. 16f). Meanwhile, the edge temperature neighboring the quartz tube was 57 K lower than the core of the pellet (Supplementary Fig. 16d), indicating that heat transfer to quartz tube decreased the temperature of the catalyst pellet side. When the CH was used, there was no significant temperature gradient (Supplementary Figure 17).


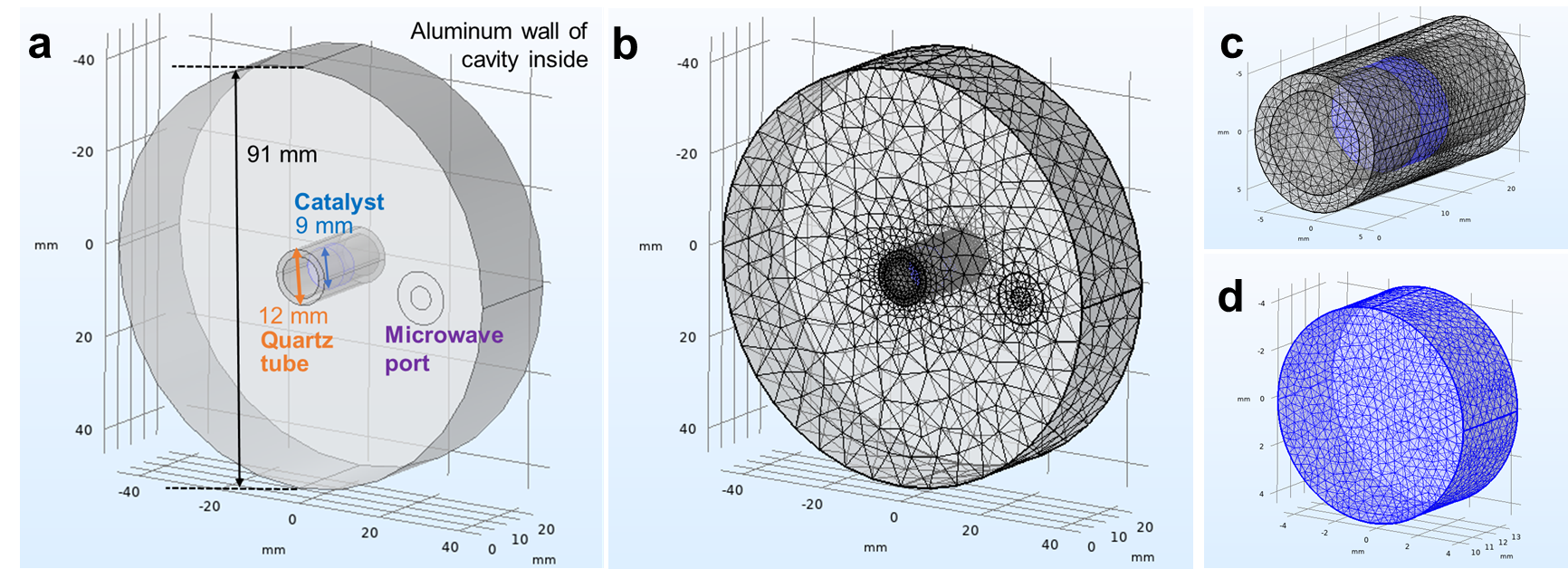


**Supplementary Fig. 14 Simulation models for determination of temperature distribution in catalyst pellet by COMSOL Multiphysics. a** Simulation model of the catalyst bed in a quartz tube and the TM_010_-mode cavity. Meshes of **b** all simulated model, **c** quartz tube model, and **d** catalyst bed model.

**Supplementary Table 7.** Physical properties of the catalysts used for COMSOL Multiphysics simulation.

| Sample | Catalyst bed  height (mm]^a^ | Dielectric constant ^a^ | | Heat capacity at  constant pressure:  C_p_ [ J/(kg·K)] ^b^ | Density:  d (kg/m^3^) ^a^ | Thermal  conductivity:  κ [W/(m·K)] ^c^ |
| --- | --- | --- | --- | --- | --- | --- |
|  |  | *ε*’ | *ε*’’ |  |  |  |
| Pt/Al_2_O_3_ | 4 | 1.5 | 0.03 | 900 | 179 | 0.29 |
| Pt/SiO_2_ | 6 | 1.3 | 0.01 | 700 | 118 | 0.20 |

^a^ Experimental value. ^b^ Value from COMSOL database. ^c^ Simulation value which reproduces experimental result.


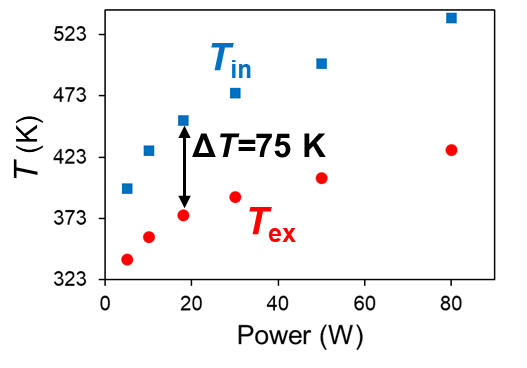


**Supplementary Fig. 15 MW heating profiles of *T*_ex_ and *T*_in_ of Pt/Al_2_O_3_ catalyst pellet under MW heating**.


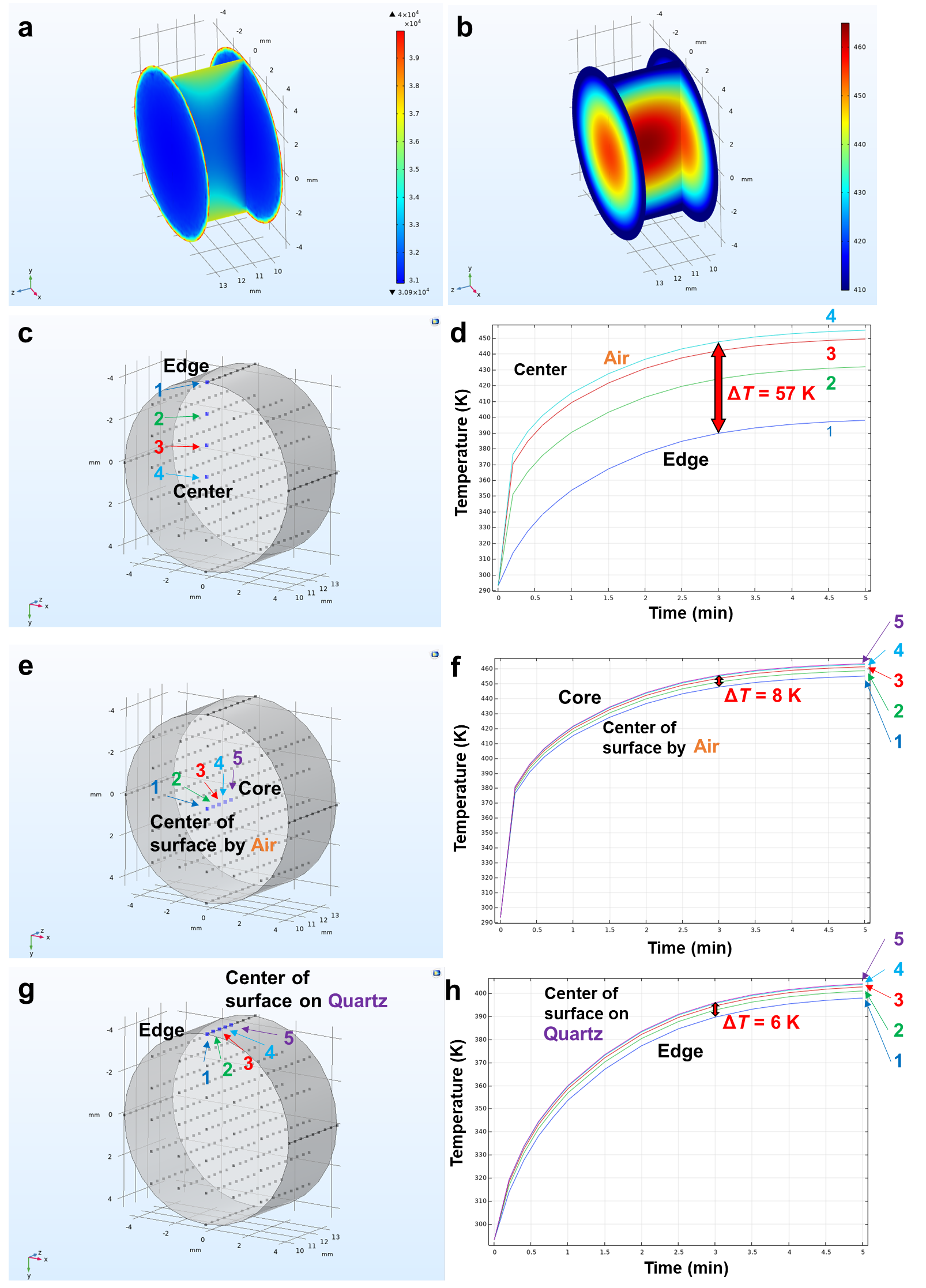


**Supplementary Fig. 16 The simulated temperature of Pt/Al_2_O_3_ catalyst pellet under 18 W MW heating.** Distributions of **a** electric field and **b** temperature. **c** Model and **d** temperature change of 4 points at the pellet surface by air. **e** Model and **f** temperature change of 5 points of an internal part of a pellet. **g** Model and **h** temperature change of 5 points at the pellet surface on the quarts tube.


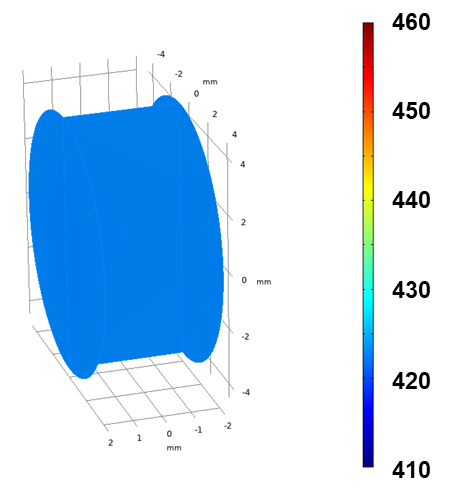


**Supplementary Fig. 17 Temperature distribution of Pt/Al_2_O_3_ under conventional heating.**

As for Pt/SiO_2_ pellet, the effective thermal conductivity of the pellet was determined as 0.20 W/(m·K) by the same method detailed above. Then, *T*_ex_ of 376 K was corrected as 471 K of *T*_in_ under 58 W MWs (Supplementary Fig. 18). The difference in *T*_Pt_ (603 K, Fig. 5f) and the *T*_in_ at the same area of Pt/SiO_2_ attained 132 K, which was 5.1-fold higher than the Pt/Al_2_O_3_. Therefore, the Pt/SiO_2_ forms larger local heating at Pt NPs by MWs than Pt/Al_2_O_3_.


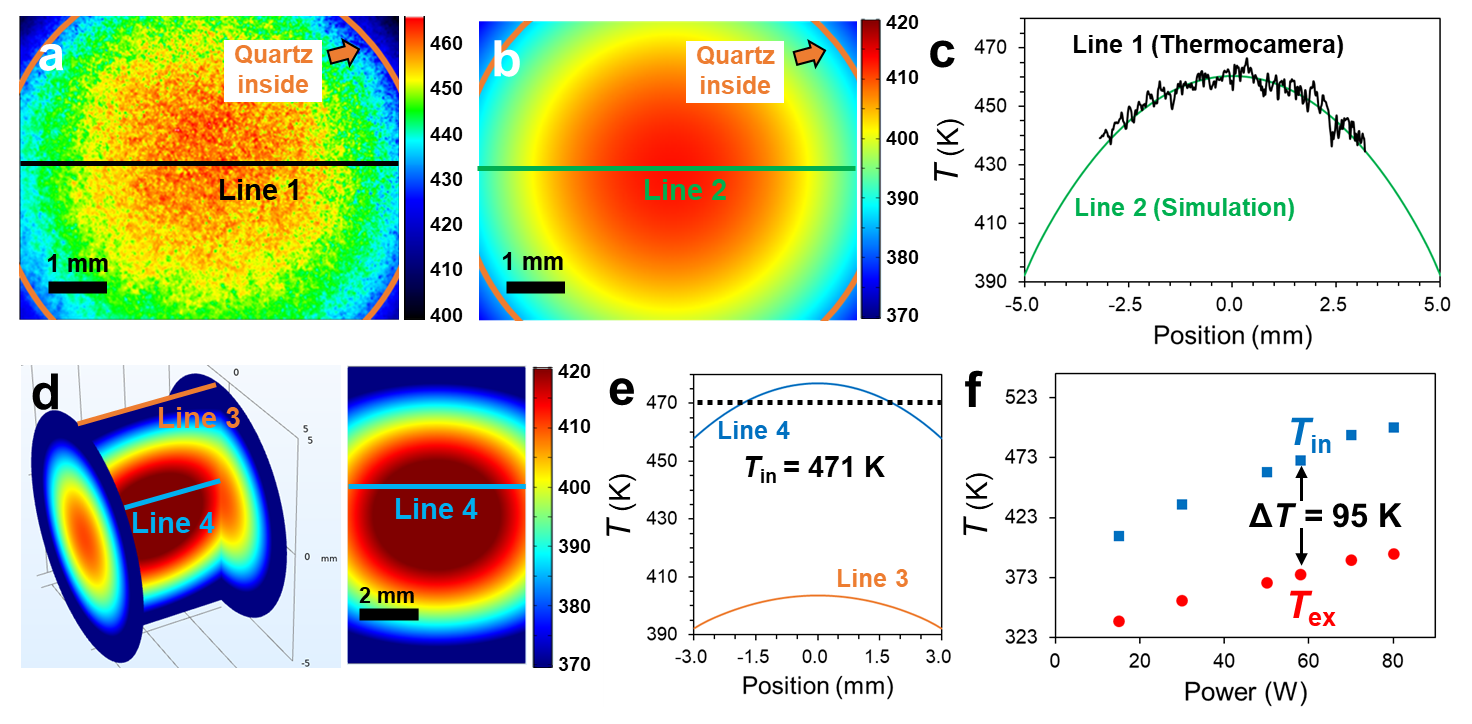


**Supplementary Fig. 18 Determination of temperature distribution of Pt/SiO_2_ catalyst pellet under MW heating. a** Temperature mapping by thermography under 58-watt MWs. **b** Reproduced temperature mapping by COMSOL Multiphysics simulation. **c** Temperature profiles at lines 1 and 2. **d** Simulational temperature mapping from a different angle. **e** Temperature profiles of lines 3 and 4. The average temperature of line 4 along with the X-ray beam was calculated as *T*_in_. **f** MW heating profiles of *T*_ex_ measured by IR thermometer and *T*_in_.

**Supplementary Note 4: Catalytic Conversion of 2-Propanol**

The dehydrogenation and dehydration reaction of 2-propanol catalyzed by Pt/Al_2_O_3_ was conducted at 373 K, 398 K, 423 K, and 448 K (Supplementary Fig. 18). The consistent yields and the selectivity in products were obtained, showing that the catalyst was stable during the reaction. Acetone was the main product generated by the dehydrogenation of 2-propanol by Pt/Al_2_O_3_. The acetone yield at 373 K was 9.6% under CH while that under MW was 16.5%. Thus, the reaction enhancement by MWs attained 1.7 times at the same temperature. The acetone yield at 398 K by CH attained 22.0%. Therefore, MW affords the lowering temperature by ~25 K for the dehydrogenation reaction of 2-propanol. The dehydration of 2-propanol also generated propylene and diisopropyl ether at a higher temperature, and MWs also enhanced the production yields.


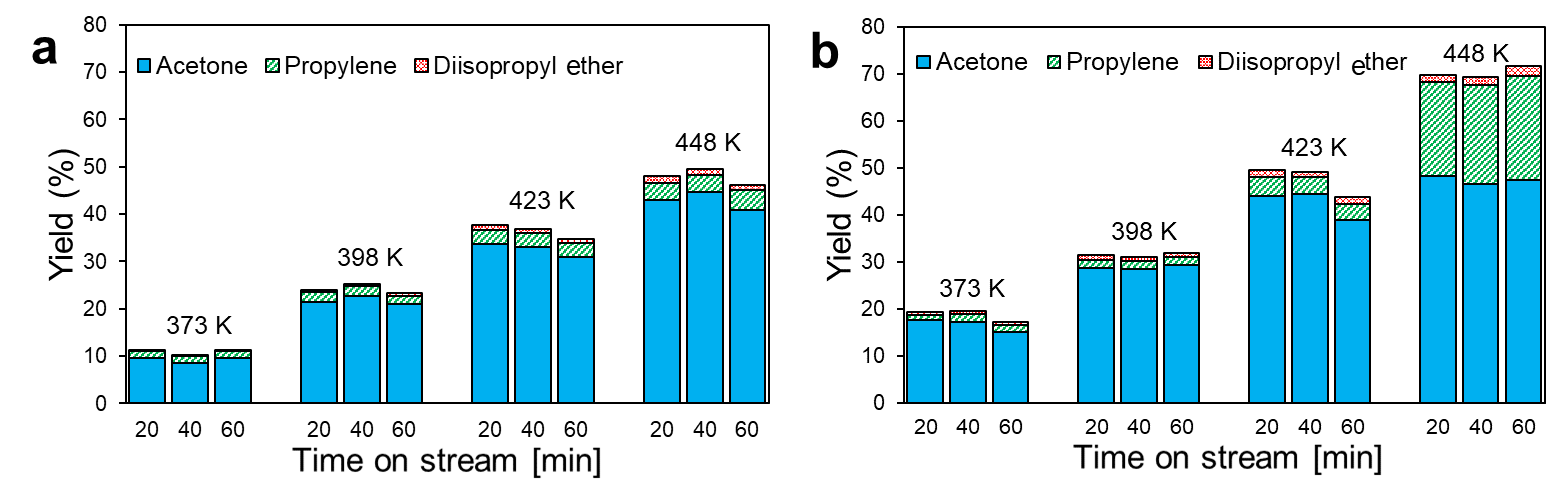


**Supplementary Fig. 19. Product distribution by catalytic conversions of 2-propanol with Pt/Al_2_O_3_ catalyst.** Under **a** CH and **b** MW heating.

Since the dehydration reaction also proceeded at the surface of the Al_2_O_3_ support, the same reaction was also tested using Al_2_O_3_ without Pt (Supplementary Fig. 20). The dehydrogenation of 2-propanol also underwent without Pt, however, the product yields were much smaller than that with Pt. The yield of diisopropyl ether by MWs at 373 K (0.64%) was larger than that by CH at 448 K (0.51%), where the intermolecular dehydration reaction of 2-propanol was enhanced. Further, the propylene from the intramolecular dehydration was produced only in the MW condition. Therefore, the dehydration reactions were dramatically enhanced in the Al_2_O_3_ catalyst. Since the γ-Al_2_O_3_ contains many defects at the surface, the microwaves were efficiently absorbed to enhance the reaction.


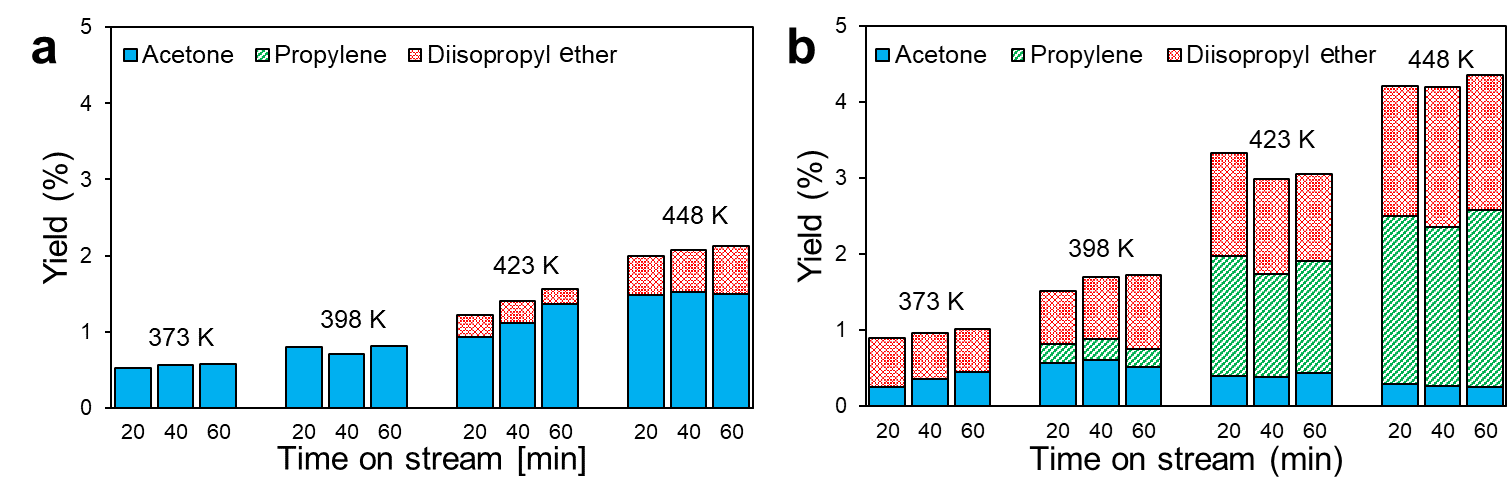


**Supplementary Fig. 20** **Product distribution by catalytic conversions of 2-propanol with Al_2_O_3_.** Under **a** CH and **b** MW heating.

Supplementary Fig. 21 summarizes the distributions of products from 2-propanol by the Pt/SiO_2_ catalyst. The acetone yield by the dehydrogenation of 2-propanol by Pt/SiO_2_ at 373 K was 2.3% under CH while that under MW attained 5.9 %, which was 2.6 times larger than CH. The acetone yields by MW at 373 K was comparable to that by CH at 398 K (3.6%) and 423 K (7.2%). Therefore, the reaction enhancement by MWs corresponds to ~50 K. Besides, the dehydration of 2-propanol proceeded above 398 K. The yields of propylene and diisopropyl ether under MW heating at 398 K were comparable to those under CH at 423 K and 448 K. Thus, the MWs reduces the reaction temperature by ~50 K. Comparing Figs. 2f and 5f, the extent of the local heating of the Pt NPs on SiO_2_ was larger than Pt/Al_2_O_3_. Therefore, we concluded that the significant local heating of Pt NPs brought the large acceleration in dehydrogenation reaction by Pt/SiO_2_. The by-products of propylene and diisopropyl ether were also increased by Pt/SiO_2_ under MW heating, implying the local high temperature of Pt NPs also facilitate the dehydration reaction of 2-propanol.


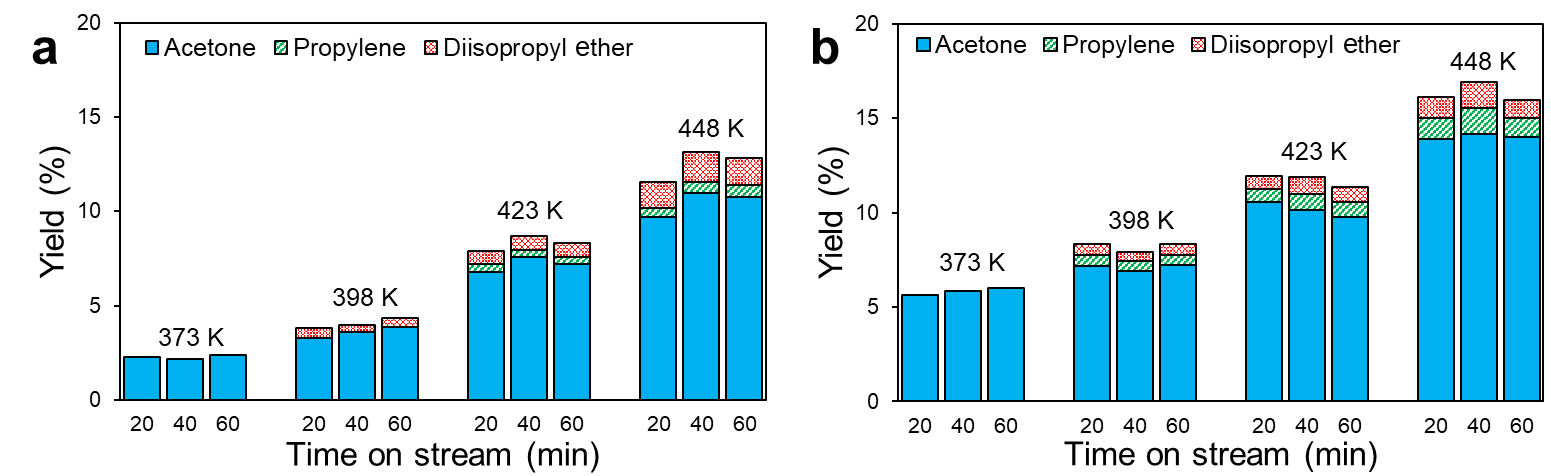


**Supplementary Fig. 21 Product distribution by dehydrogenation and dehydration reactions of 2-propanol catalyzed by Pt/SiO_2_.** Under **a** CH and **b** MW heating.

The inner temperatures of the catalyst bed were also measured by the fiber optic thermometer to evaluate the effect of temperature distribution in the packed catalyst bed in the macro-scale (Supplementary Fig S22). There were no significant temperature differences between *T*_ex_ and *T*_core_ between Pt/Al_2_O_3_ and Pt/SiO_2_. Therefore, the larger acceleration in Pt/SiO_2_ catalyst can be explained by the local heating of the Pt NPs.


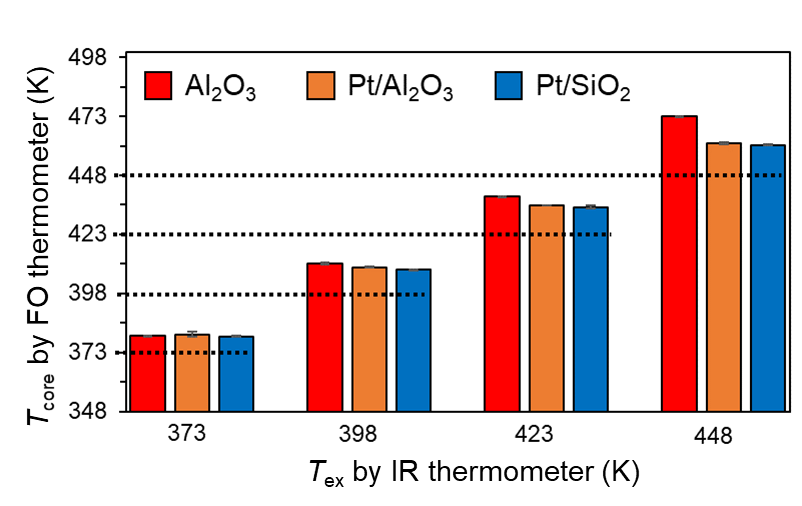


**Supplementary Fig. 22 The core temperature of the catalyst beds (*T*_core_) during dehydrogenation reaction of 2-propanol under MW heating.** *T*_core_ was measured by a fiber-optic thermometer as compare to the surface temperature measured by the IR thermometer (*T*_ex_).
